# The Origin and Evolution of Plastid Genome Downsizing in Southern Hemispheric Cypresses (Cupressaceae)

**DOI:** 10.3389/fpls.2020.00901

**Published:** 2020-06-23

**Authors:** Edi Sudianto, Chung-Shien Wu, Shu-Miaw Chaw

**Affiliations:** Biodiversity Research Center, Academia Sinica, Taipei, Taiwan

**Keywords:** callitroids, Callitroideae, cupressophytes, plastid genome, genome downsizing, Southern Hemisphere

## Abstract

Plastome downsizing is rare in photosynthetic seed plants. However, a large-scale study of five cupressophyte families (conifers II) indicated that the plastomes of some Cupressaceous genera are notably reduced and compact. Here, we enriched taxon sampling in Cupressaceae by adding plastomes of ten previously unreported genera to determine the origin, evolution, and consequences of plastome reduction in this family. We discovered that plastome downsizing is specific to Callitroideae (a Southern Hemispheric subfamily). Their plastomes are the smallest, encode the fewest plastid genes, and contain the fewest GC-end codons among Cupressaceae. We show that repeated tRNA losses and shrinkage of intergenic spacers together contributed to the plastome downsizing in Callitroideae. Moreover, our absolute nucleotide substitution rate analyses suggest relaxed functional constraints in translation-related plastid genes (*clpP, infA, rpl*, and *rps*), but not in photosynthesis- or transcription-related ones, of *Callitris* (the most diverse genus in Callitroideae). We hypothesize that the small and low-GC plastomes of Callitroideae emerged ca. 112–75 million years ago as an adaptation to increased competition with angiosperms on the Gondwana supercontinent. Our findings highlight Callitroideae as another case of plastome downsizing in photosynthetic seed plant lineages.

## Introduction

Cupressaceae (cypress family) is the most widely distributed family in cupressophytes (conifers II), comprising 30–32 genera and ca. 133 species. Many members of the family are economically and ecologically important. For example, bald cypress (*Taxodium*), China fir (*Cunninghamia*), cordilleran cypress (*Austrocedrus*), and cypress pine (*Callitris*) are important sources of timber, furniture, and ornamentals (Farjon, 2005). They are distributed across all continents except Antarctica (Farjon, 2005; Brodribb et al., 2012). Cypress genera have been classified into seven (or six) subfamilies (Mao et al., 2012; Yang et al., 2012). Two of the seven subfamilies, Athrotaxidoideae and Callitroideae, are mainly restricted to the Southern Hemisphere, while the other five (Cupressoideae, Cunninghamioideae, Sequoioideae, Taiwanioideae, and Taxodioideae) are mostly distributed in the Northern Hemisphere (Gadek et al., 2000; Farjon and Filer, 2013).

Farjon (2005) maintained that there are only six Cupressaceous subfamilies. He merged Callitroideae into Cupressoideae and ranked the Southern Hemispheric clade as the Callitrideae tribe. However, most molecular phylogenetic studies consider the subfamily Callitroideae as separate from Cupressoideae (Leslie et al., 2012; Mao et al., 2012; Yang et al., 2012). Callitroideae is the least abundant conifer group (Leslie et al., 2012); most (ca. 60%) of its genera are monotypic, except for *Callitris*, which has more than 10 recognized species (Farjon, 2005). In addition, previous studies on the phylogenetic positions of early-diverging Callitroideae genera (e.g., *Austrocedrus, Pilgerodendron*, and *Libocedrus*) have been inconsistent and require further scrutiny (Mao et al., 2012; Yang et al., 2012; Crisp et al., 2019).

Plastid genomes (plastomes) of seed plants are generally conserved in both gene order and content. The average size of photosynthetic seed plant plastomes is ∼145 kb (Jansen and Ruhlman, 2012), but exceptions have been reported in gnetophytes (109–119 kb; Wu et al., 2009), pines (107–122 kb; Lin et al., 2010; Sudianto et al., 2016), saguaro cactus (∼113 kb; Sanderson et al., 2015), cupressophytes (121–136 kb; Wu and Chaw, 2016), and legumes (∼121 kb; Choi et al., 2019). Most of these size reductions were caused by the loss of one copy of inverted repeats (IRs), non-coding DNA, or non-essential plastid genes. Some commonly lost plastid genes are the NADH dehydrogenase-like complex (*ndh*), various small and large ribosomal proteins (*rps* and *rpl*), and several transfer RNA (tRNA) genes (Wu et al., 2009; Sanderson et al., 2015; Chaw et al., 2018).

Photosynthetic seed plant plastomes typically encode 29–32 tRNA genes (Jansen and Ruhlman, 2012), far fewer than the 61 standard sense codons. To account for this disparity, Crick (1966) proposed the “wobble hypothesis,” which posits that a minimum of 32 tRNA species is needed to read all codons. This hypothesis suggests that the third codon position can have a non-Watson-Crick base-pair interaction with the first position of the tRNA anticodon (Crick, 1966). More recently, however, studies on tobacco plastomes further narrowed the minimum set of essential tRNA genes down to 25 tRNA species, calling it the “superwobble hypothesis” (Alkatib et al., 2012a, b).

Yet, *Callitris* has been documented to have lost some of the essential tRNA genes, including *trnG-UCC, trnT-UGU*, and *trnV-UAC* (Wu and Chaw, 2016). The *Callitris* plastome – the smallest, most compact, and most rearranged plastome in the Cupressaceae (Wu and Chaw, 2016; Chaw et al., 2018) – also shows several unique characteristics that highlight major evolutionary events occurring after *Callitris* diverged from its sister clade. However, it remains unknown whether these features are specific to *Callitris* or common to all Southern Hemispheric Cupressaceous genera. Thus, extending taxon sampling to include more Callitroideae genera will offer critical information on the origin, evolution, and implications of the above-mentioned features.

Here, we increase sampling of Cupressaceous plastomes by adding ten previously unreported genera, including six from Callitroideae (8–10 recognized genera), two from Cupressoideae (11–13 recognized genera), and one each from Athrotaxidoideae (one recognized genus) and Sequoioideae (three recognized genera). These new sequences fill in the gaps in our understanding of plastomic variation in Cupressaceae, especially among the Southern Hemispheric lineages. Specifically, we address the following questions: (1) Do all Southern Hemispheric Cupressaceous genera commonly have reduced plastomes and fewer plastid tRNAs? and (2) Do losses of many tRNAs influence the codon compositions or nucleotide substitution rates in the plastomes of Callitroideae?

## Materials and Methods

### Sample Collection and DNA Extraction

Studied samples were collected from trees growing in the University of California Botanical Garden (Berkeley, United States) and Botanischer Garten der Heinrich-Heine-Universität (Düsseldorf, Germany). The specimens were deposited into the Herbarium of Academia Sinica, Taipei, Taiwan (HAST). Collection information, voucher numbers, and GenBank accession numbers are listed in Supplementary Table S1. We extracted total genomic DNA from each sample following a modified CTAB method (Stewart and Via, 1993).

### Sequencing, Assembly, and Annotation

Total DNA was sequenced on an Illumina NextSeq500 platform at Genomics BioSci & Tech (New Taipei City) or Tri-I Biotech (New Taipei City), generating ∼2–4 Gb of paired-end (2 × 150 bp) reads for each species. Trimmomatic 0.36 (Bolger et al., 2014) and FastQC 0.11.5 (Andrew, 2011) were used to trim and check the quality of the reads, respectively. Plastomes were *de novo* assembled using Ray 2.3.1 (Boisvert et al., 2010) and plastid contigs/scaffolds were BLAST-searched using the *Taiwania flousiana* plastome (NC_021441) as the reference with an *E-*value of <10^–10^. We obtained complete plastomes for most of the sampled taxa using this method. However, plastomic assemblies were fragmented in a few taxa. We therefore used these fragmented assemblies and NOVOPlasty 2.7.2 (Dierckxsens et al., 2017) to generate complete plastomes. Plastome annotation was conducted using the “Transfer Annotation” function in Geneious 11.0.5 (Kearse et al., 2012) with the *T. flousiana* plastome as the reference, followed by manual corrections. Transfer RNA (tRNA) genes were further confirmed using tRNAscan-SE 2.0 (Lowe and Chan, 2016). Repetitive sequences were identified using REPuter (Kurtz et al., 2001) with a minimum size of 8 bp.

### Sequence Alignment and Phylogenetic Tree Reconstruction

We extracted 79 common plastid protein-coding genes from 29 Cupressaceous genera (including 10 newly sequenced in this study) and five closely related genera (one Sciadopityaceae and four Taxaceae). Each gene was aligned using MUSCLE (Edgar, 2004) implemented in MEGA 7.0 (Kumar et al., 2016) with the “Align Codon” option. SequenceMatrix (Vaidya et al., 2011) was used to concatenate the alignment. A matrix with 64,185 nucleotide sites was obtained and used to reconstruct the maximum likelihood (ML) and Bayesian inference (BI) trees using RAxML 8.2.10 (Stamatakis, 2014) and MrBayes 3.2.6 (Huelsenbeck and Ronquist, 2001), respectively, using the GTR + G + I model, as suggested by jModelTest 2.1.10 (Darriba et al., 2012) and MrModelTest 2.4 (Nylander, 2004). The ML tree was assessed from 1,000 bootstrap replicates, and we ran two independent analyses for the BI tree, using 6,000,000 generations for each run and sampling every 300 generations. We discarded the first 25% of the samples as burn-in, assessed the level of convergence (convergence level increases as the Potential Scale Reduction Factor score approaches 1), and ensured that the average standard deviation of split frequencies was below 0.01. To infer the tRNA gene gain/loss history, we performed Dollo’s parsimony using Count (Csurös, 2010) and topology inferred from phylogenetic analyses.

### Ancestral Plastome Reconstruction

We identified the locally collinear blocks (LCBs) between the 34 sampled genera and *Cycas taitungensis* (AP009339) plastomes using progressiveMauve (Darling et al., 2010). The IR_A_ region of the *Cycas* plastome was removed prior to the analysis because previous studies indicated that cupressophyte plastomes lost their IR_A_ (Wu et al., 2011). The ancestral plastomes were reconstructed based on the LCB order matrices and ML tree topology in MLGO (Hu et al., 2014). Subsequently, the reconstructed ancestral plastomes were compared with their close descendants to infer the plastomic inversion history across the phylogeny in GRIMM (Tesler, 2002).

### Codon Usage Bias Calculation

We calculated relative synonymous codon usage (RSCU) scores from 61 non-stop codons in the 79 common plastid protein-coding genes of 29 Cupressaceous, one Sciadopityaceous, and four Taxaceous genera using DAMBE 7.0.58 (Xia, 2018). Next, we performed correspondence analyses on the RSCU values using the FactoMineR package (Lê et al., 2008) in R 3.6.1 (R Core Team, 2019). The 34 genera were further classified into five groups based on the hierarchical clustering of the 61 RSCU values.

### Estimation of Nucleotide Substitution Rates and Molecular Dating

**FIGURE 1:**
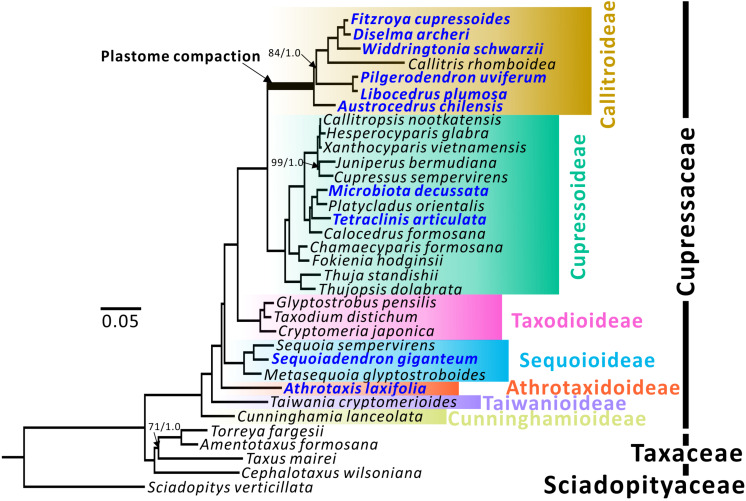
Plastid phylogenomic trees of seven Cupressaceous subfamilies. *Sciadopitys verticillata* was designated as the outgroup. The tree framework is based on the ML tree. All nodes received full 100% bootstrap support (BS) or 1.0 posterior probability (PP) from ML/BI analyses, except for the three with BS/PP values. Newly sequenced species are in blue.

Non-synonymous (*d*_*N*_) and synonymous substitution rates (*d*_*S*_) of the plastid genes were estimated using the CODEML program in PAML 4.9i (Yang, 2007) with the following parameters: runmode = 0, seqtype = 1, CodonFreq = 2, estFreq = 0, model = 1, and cleandata = 1. The constrained tree topology was obtained from the ML analysis (Figure 1). Divergence times were estimated using the approximate likelihood method in MCMCTREE (dos Reis and Yang, 2011) in PAML 4.9i. We used six estimated points from TimeTree (Hedges et al., 2015) as the calibrated ages of six specific nodes (Supplementary Figure S4). MCMC sampling was run for 2,500,000 generations and samples were drawn every 500 generations, with a burn-in of 200,000. We performed two independent runs to evaluate convergence. Tracer 1.7 (Rambaut et al., 2018) was used to confirm that the effective sample sizes of all parameters were above 200. Absolute substitution rates were calculated by dividing the *d*_*N*_ and *d*_*S*_ branch lengths over their respective divergence times. Only terminal branch lengths were considered in this study. The branch-site model test was performed using EasyCodeML (Gao et al., 2019).

## Results

### Support for Seven Distinct Cupressaceous Subfamilies

Topologies of maximum likelihood (ML) and Bayesian inference (BI) trees inferred from 79 concatenated protein-coding genes have 100% bootstrap support (BS) and 1.0 posterior probability (PP) at almost all nodes (Figure 1). Among the seven subfamilies, Cunninghamioideae is the earliest diverging clade, followed by Taiwanioideae, Athrotaxidoideae, Sequoioideae, and Taxodioideae. Taxodioideae is sister to a clade encompassing Cupressoideae and Callitroideae. In Callitroideae, the genus *Austrocedrus* diverged first and the *Pilgerodendron*–*Libocedrus* clade is sister to the clade containing four genera: *Fitzroya–Diselma, Widdringtonia*, and *Callitris*. Notably, the branch leading to Callitroideae is remarkably long and contains a signature of positive selection (branch-site model: *P* < 0.001), suggesting that positive selection resulted in accelerated rates of plastid nucleotide substitutions before the diversification of Callitroideae. Hence, the Southern Hemispheric clade should be treated as a separate subfamily from Cupressoideae.

### Callitroideae Has the Smallest and Most Rearranged Plastomes in Cupressaceae

Table 1 compares the newly sequenced plastomes from ten Cupressaceous genera: one Athrotaxidoideae, six Callitroideae, two Cupressoideae, and one Sequoioideae. Their GC contents were similar (34.0–35.5%), but coding gene numbers varied, particularly with tRNA. For example, we detected 28–31 plastid tRNAs in Callitroideae but 33–34 in other Cupressaceous subfamilies (Table 1). In addition, the monotypic species *Austrocedrus chilensis* (Callitroideae) lacked *psaM*. This species thus has fewer plastid protein-coding genes than any taxa studied so far. Overall, plastomes of Callitroideae were relatively gene-poor compared to other subfamilies (114–116 genes, compared to 119–120 elsewhere).

**TABLE 1:** Plastomic features of the ten newly sequenced Cupressaceous species.

		Plastome					Total no.	Non-genic		
Species		size (bp)	GC (%)	No. of genes			of genes	content (%)		Repeat (%)
				Protein-coding	rRNA	tRNA		Intron	IGS	
Athrotaxidoideae	*Athrotaxis laxifolia*	132,660	34.4	82	4	33	119	8.6	29.1	4.6
Sequoioideae	*Sequoiadendron giganteum*	131,472	35.5	82	4	34	120	8.8	30.4	3.5
Callitroideae	*Austrocedrus chilensis*	123,133	34.7	81	4	31	116	8.9	25.5	3.7
	*Diselma archeri*	124,251	34.5	82	4	30	116	8.8	26.4	5.1
	*Fitzroya cupressoides*	123,548	34.6	82	4	30	116	8.8	25.2	4.8
	*Libocedrus plumosa*	121,344	34.1	82	4	28	114	8.3	25.4	4.3
	*Pilgerodendron uviferum*	121,839	34.0	82	4	28	114	8.2	25.9	5.1
	*Widdringtonia schwarzii*	123,190	34.3	82	4	30	116	8.3	25.4	7.4
Cupressoideae	*Microbiota decussata*	127,230	34.8	82	4	33	119	9.0	26.5	3.3
	*Tetraclinis articulata*	125,797	34.9	82	4	33	119	9.0	27.0	3.8

*IGS, Intergenic spacers.*

**FIGURE 2:**
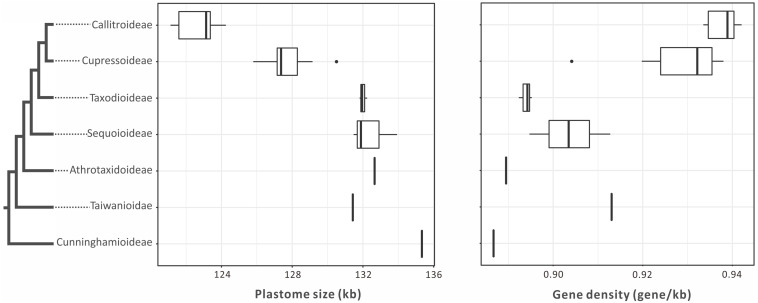
Boxplots for comparing plastome size and gene density among seven Cupressaceous subfamilies. The plastomes of Callitroideae are the smallest and most compact. The black dots represent outliers. The phylogenetic backbone is based on Figure 1.

Plastomes are generally smaller in Callitroideae (121,344–124,251 bp) than other subfamilies (125,797–132,660 bp). An analysis of their introns, intergenic spacers (IGS), and repeats indicates that Callitroideae has lower IGS content (25.2–26.4%) than other subfamilies (26.5–30.4%) but similar intron and repeat content (Table 1). Thus, the shrinkage of IGS resulted in the reduction of plastid non-coding regions in Callitroideae. To create a better picture of the evolution of plastome size in Cupressaceae, available taxa from all living subfamilies were included and compared. As shown in Figure 2, Callitroideae plastomes are significantly smaller and more compact (gene-dense) than those of other subfamilies (Mann-Whitney test; all *P* < 0.01). Moreover, plastomic rearrangements were the most extensive in Callitroideae (Supplementary Figure S1). We detected three rearrangement hotspots: seven in *Callitris*, five in the common ancestor of *Libocedrus* and *Pilgerodendron*, and five rearrangements in the common ancestor of all Callitroideae genera (Supplementary Figure S1). Together, these results show that Callitroideae plastomes are not only the smallest but also the most rearranged among Cupressaceae.

### Callitroideae Plastomes Repeatedly Lost tRNAs and Have Distinct Codon Compositions

**FIGURE 3:**
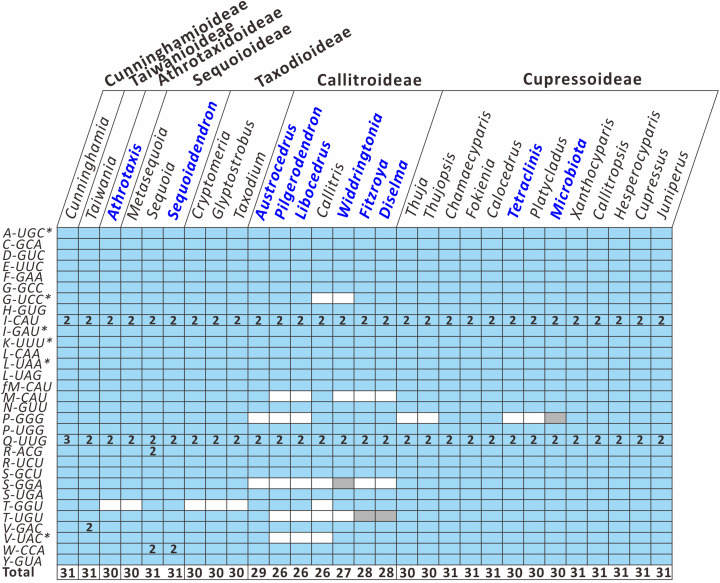
Comparison of plastid tRNA gene repertoires among seven Cupressaceous subfamilies. The functional copy, pseudogene copy, and gene loss are denoted in blue, gray, and white, respectively. Arabic numerals within the boxes indicate the number of gene copies. The total number of distinct tRNA species for each genus is listed in the bottom row. Newly sequenced plastomes are in dark blue font. Intron-containing genes are marked with an asterisk (*).

To better understand how the plastid tRNA gene repertoire changes across the Cupressaceae, we compared 29 available genera in the seven Cupressaceous subfamilies and found that all genera had 30–31 tRNA species – except for the seven Callitroideae genera, which had only 26–29 tRNA species (Figure 3). Callitroideae genera lost various plastid tRNAs, including *trnG-UCC, trnM-CAU, trnP-GGG, trnS-GGA, trnT-GGU, trnT-UGU*, and *trnV-UAC* (Figure 3). Our analysis further supports the assertion that 16 plastid tRNA loss events occurred across Cupressaceae (Supplementary Figure S2). Several tRNAs were repeatedly lost from different lineages. For example, during the evolution of Cupressaceae, there were multiple independent losses of *trnG-UCC* (2 times), *trnM-CAU* (2 times), *trnP-GGG* (3 times), *trnT-GGU* (4 times), and *trnV-UAC* (2 times). The majority (10) of these tRNA losses occurred within Callitroideae, suggesting that some driving force differentiates Callitroideae from other subfamilies in the retention of plastid tRNAs.

**FIGURE 4:**
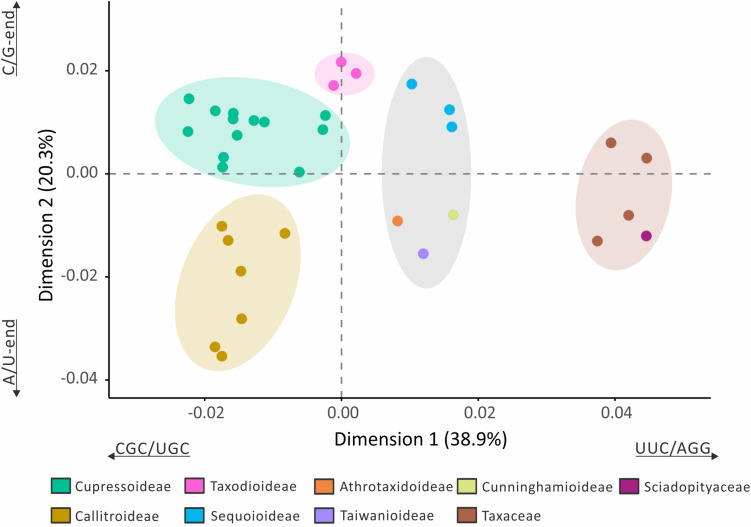
Correspondence analysis of plastid relative synonymous codon usage (RSCU). Thirty-four genera were included in the analysis (29 Cupressaceae and five closely related genera: one Sciadopityaceae and four Taxaceae). Hierarchical clustering clearly reveals five separate groups. The first two dimensions accounted for 59.2% of the RSCU variation among the 34 sampled genera.

The loss of many plastid tRNA genes from the Callitroideae prompted us to investigate whether this subfamily has a distinct codon composition. We performed a correspondence analysis (CA) using 61 non-stop relative synonymous codon usage (RSCU) scores from 29 Cupressaceous genera and five genera from two closely related families [Sciadopityaceae (1) and Taxaceae (4)] as the outgroup for comparison. The first two CA dimensions predicted about 59.2% of the RSCU variation. Hierarchical clustering resolved five isolated groups in the 34 sampled genera (Figure 4). The first dimension (38.9%) clearly distinguished Cupressaceae from Sciadopityaceae and Taxaceae, with CGC, UGC, UUC, and AGG codons constituting the largest contributions. The second dimension explained about 20.3% of the RSCU variation and separated Callitroideae from its sister subfamily, Cupressoideae. Our analyses further indicate that this separation is mainly caused by nucleotides at the 3rd codon position – i.e., Callitroideae plastomes have more A/U-ending codons, while Cupressoideae plastomes contain more C/G-ending codons (Figure 4). Moreover, Callitroideae and Athrotaxidoideae plastomes contain the lowest number of GC nucleotides, especially at the 3rd codon position (Supplementary Figure S3). No evidence of plastid codon reassignments was detected in Callitroideae (Supplementary Table S3).

### Relaxed Functional Constraints on Plastid Translation-Related Genes in *Callitris*

**FIGURE 5:**
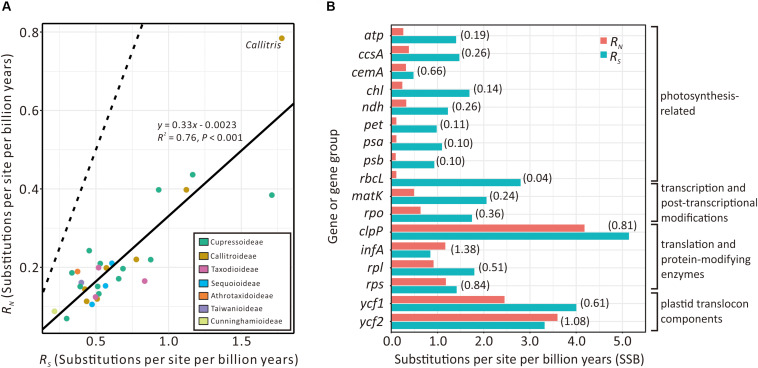
Comparison of plastid absolute non-synonymous (*R*_*N*_) and synonymous (*R*_*S*_) substitution rates across seven Cupressaceous subfamilies. **(A)** Regression analysis showing a strong positive correlation between *R*_*S*_ and *R*_*N*_ among the 29 sampled genera. The solid line depicts the linear regression line with its equation, *R*^2^, and *P*-value indicated. The dashed line indicates the expected pattern if *R_*S*_* = *R*_*N*_. **(B)** Bar plot showing *R*_*N*_ vs. *R*_*S*_ of plastid genes/groups in *Callitris*. *R_*N*_/R_*S*_* ratios are in parentheses.

Figure 5A compares the absolute synonymous (*R*_*S*_) and non-synonymous (*R*_*N*_) substitution rates estimated from the 29 sampled Cupressaceous genera. *R*_*S*_ and *R*_*N*_ are strongly and positively correlated (*R*^2^ = 0.76; *P* < 0.001). *Callitris* exhibits the highest values of both *R*_*S*_ and *R*_*N*_, noticeably deviating from the expected values based on the regression line. We also detected elevated *R*_*N*_/*R*_*S*_ ratios in some *Callitris* genes. For example, the *R_*N*_/R_*S*_* ratios of plastid translation-related genes (*clpP*, *infA*, *rpl*, and *rps*) and plastid translocon component genes (*ycf1* and *ycf2*) were greater than 0.5. Two genes, *infA* (1.38) and *ycf2* (1.08), had *R_*N*_/R_*S*_* ratios greater than 1 (Figure 5B). In contrast, the *R_*N*_/R_*S*_* ratio of other genes, such as photosynthesis- and transcription-related genes, were generally lower than 0.4 in *Callitris*. Collectively, these results may suggest that *Callitris* encountered positive selection or relaxed functional constraints in plastid genes related to translation and protein modification.

## Discussion

### Filling the Gaps in Plastid Phylogenomics of Cupressaceae

Prior plastid phylogenomics of Cupressaceae focused on the taxa of Northern Hemispheric origin (Wu and Chaw, 2016; Qu et al., 2017a, b; Zhu et al., 2018) and only investigated one Southern Hemispheric taxon (*Callitris rhomboidea*; included in Wu and Chaw, 2016). The present study adds ten newly elucidated plastomes, including seven Southern Hemispheric taxa (six Callitroideae and one Athrotaxidoideae) and three Northern Hemispheric taxa (two Cupressoideae and one Sequoioideae). The addition of these ten genera enabled us to build the most comprehensive plastid phylogenomics of Cupressaceae to date – amounting to 29 of the 30–32 Cupressaceous genera recognized worldwide.

Both our ML and BI trees (Figure 1) support the monophyly of Callitroideae, with *Austrocedrus* sister to the remaining genera. This agrees with the views of Mao et al. (2012); Yang et al. (2012), and Leslie et al. (2018), but not Crisp et al. (2019) who placed the *Pilgerondendron–Libocedrus* clade as sister to the remaining Callitroideae genera. The phylogenetic positions of other Callitroideae genera are also congruent with the four above-mentioned studies. The intergeneric relationships within Cupressoideae and Sequoioideae agree well with those of Mao et al. (2012) and Leslie et al. (2018). *Athrotaxis*, the sole genus of Athrotaxidoideae, is supported as sister to a clade including Taxodioideae, Sequoioideae, Callitroideae, and Cupressoideae (Figure 1). This placement, however, contradicts those of Yang et al. (2012) and Lu et al. (2014), which placed *Athrotaxis* as sister to the Taxodioideae–Callitroideae–Cupressoideae clade and the Sequoioideae subfamily, respectively.

### Numerous tRNA Gene Losses and IGS Reductions Contributed to Plastome Downsizing in Callitroideae

We show that plastome downsizing is especially pronounced in the Callitroideae genera. No plastome reduction was observed in the other Southern Hemispheric clade, Athrotaxidoideae. Callitroideae plastomes lost seven tRNA genes, several of which are dispensable. For example, *trnP-GGG* is frequently lost from the plastomes of major land plant lineages, such as mosses (*Physcomitrella patens* and *Tortula ruralis*; Oliver et al., 2010), liverworts (*Marchantia polymorpha*; Ohyama, 1996), lycophytes (*Selaginella*; Mower et al., 2019), gymnosperms (*Sciadopitys verticillata*; Hsu et al., 2016), and all angiosperms (Wicke et al., 2011). Two other genes (*trnS-GGA* and *trnT-GGU*) have been experimentally verified as non-essential plastid genes (Alkatib et al., 2012b). In contrast, the absence of any of the four remaining tRNA genes (*trnG-UCC, trnM-CAU, trnT-UGU*, and *trnV-UAC*) is lethal for tobacco (Alkatib et al., 2012a, b). It is interesting to note that some parasitic plants still retain ∼30 plastid tRNA genes despite having smaller plastomes than those of Callitroideae genera (see Wicke and Naumann, 2018).

Loss of these essential tRNA genes is unique to the Callitroideae, as no other Cupressaceous subfamilies have lost any of them (Figure 3). It was previously proposed that losses of tRNA genes might be compensated either by: (1) codon reassignment (Kollmar and Mühlhausen, 2017), (2) wobbling or superwobbling mechanisms (Alkatib et al., 2012b), or (3) cytosolic tRNA import (Maréchal-Drouard et al., 1999). Although codon reassignment has evidently rectified the missing tRNA in plastomes of several taxa (Su et al., 2019; Turmel et al., 2019), it was not detected in our data (Supplementary Table S3). Wobbling or superwobbling also could not compensate for these tRNA losses since the four missing tRNA genes are part of the minimum set of 25 tRNA species required to encode all codons (Alkatib et al., 2012b). Instead, the Callitroideae plastids may import cytosolic tRNAs to replace the tRNA defect, as previously proposed in various parasitic plants (Wolfe et al., 1992; Su et al., 2019). The exact mechanisms of essential tRNA compensation in Callitroideae plastids will be an interesting topic for further studies.

The mutational burden hypothesis posits that excess non-coding DNA is a liability because any mutation at the intronic splice sites, transcription factor binding sites, or core promoters will be deleterious to the genome (Lynch, 2006). Lineages with low mutation rates tend to accumulate non-coding DNA, leading to the assertion that elevated mutation rates enhance the degree of genome compaction in organelles (Lynch, 2006; Smith, 2016). Our results demonstrate signatures of both accelerated substitution rates and positive selection on the branch leading to Callitroideae (Figure 1). Conforming to the mutational burden hypothesis, this accelerated rate might have facilitated the removal of non-functional sequences, contributing to the low IGS content in Callitroideae (Table 1). Together, these data suggest that the plastome downsizing in the Callitroideae occurred prior to the subfamily diversification ca. 112–75 million years ago (Cretaceous Period; Supplementary Figure S4). This timeline coincides with the fragmentation of East and West Gondwana into smaller continents and the transition period from gymnosperm-dominated to angiosperm-dominated flora on the Gondwana (McLoughlin, 2001).

### Potential Implications of Reduced and Compact Plastomes in Callitroideae

Given their small plastome size, Callitroideae genera might have selective advantages over other subfamilies containing larger plastomes. Streamlined genomes are hypothesized to have shorter replication times and consume fewer resources such as phosphorus and nitrogen (Hessen et al., 2010; Mann and Chen, 2010). The saved resources can be reallocated to RNA production and increasing the rate of protein synthesis, eventually leading to a higher growth rate (Hessen et al., 2010). Similarly, several studies also linked genome size to cell size, arguing that smaller genomes may facilitate cell size reduction, and consequently faster growth (Bennett, 1987; Hessen et al., 2010; Simonin and Roddy, 2018). However, the trend is less straightforward in algal plastomes, not all of which have a strong positive correlation between plastome size and cell size (Smith, 2017). As plastome downsizing has been commonly found in various lineages, it may be worthy to investigate the correlation among plastome size, cell size, and growth rate.

Callitroideae plastomes also contain the fewest GC-end codons among the Cupressaceae subfamilies (Figure 4 and Supplementary Figure S3). This bias in the base composition might stem from the fact that GC nucleotides are energetically more “expensive” than AT nucleotides (Rocha and Danchin, 2002). The AT base-pair also uses one fewer nitrogen atom than the GC base-pair (Dufresne et al., 2005). Collectively, these imply that the downsized plastomes of Callitroideae may have a competitive edge over other sympatric plants. These characteristics closely resemble the plastomes of another gymnosperm group, the gnetophytes, which grow well in arid or angiosperm-dominated environments (Wu et al., 2009).

## Data Availability Statement

The complete plastid genomes of the ten Cupressaceous genera have been deposited in the DDBJ under the accession numbers LC500575–LC500584.

## Author Contributions

ES performed the experiments, analyzed the data, and wrote the manuscript. S-MC collected the plant specimens and supervised the project. C-SW and S-MC critically revised and edited the manuscript. All authors contributed to the article and approved the submitted version.

## Conflict of Interest

The authors declare that the research was conducted in the absence of any commercial or financial relationships that could be construed as a potential conflict of interest.
