# Supplementary material for: The Origin and Evolution of Plastid Genome Downsizing in Southern Hemispheric Cypresses (Cupressaceae)
Source: Front Plant Sci. 2020 Jun 23;11:901. doi: 10.3389/fpls.2020.00901 (PMC7324783; doi:10.3389/fpls.2020.00901)
Supplement: Supplementary file 1 [file Data_Sheet_1.PDF]

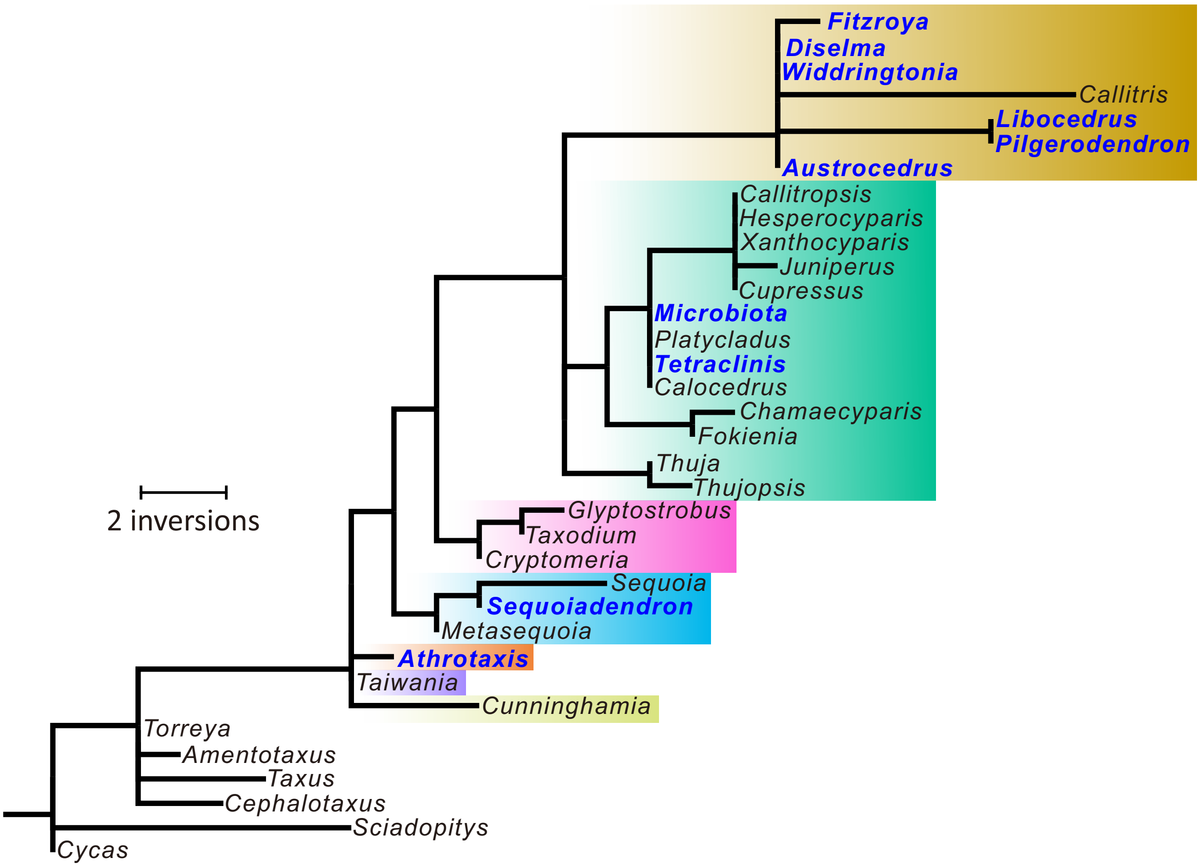

Figure S1. Inferred plastomic inversions during the evolution of Cupressaceae. Branch lengths are scaled by inversion numbers. The phylogeny backbone is the same as in Figure 1.

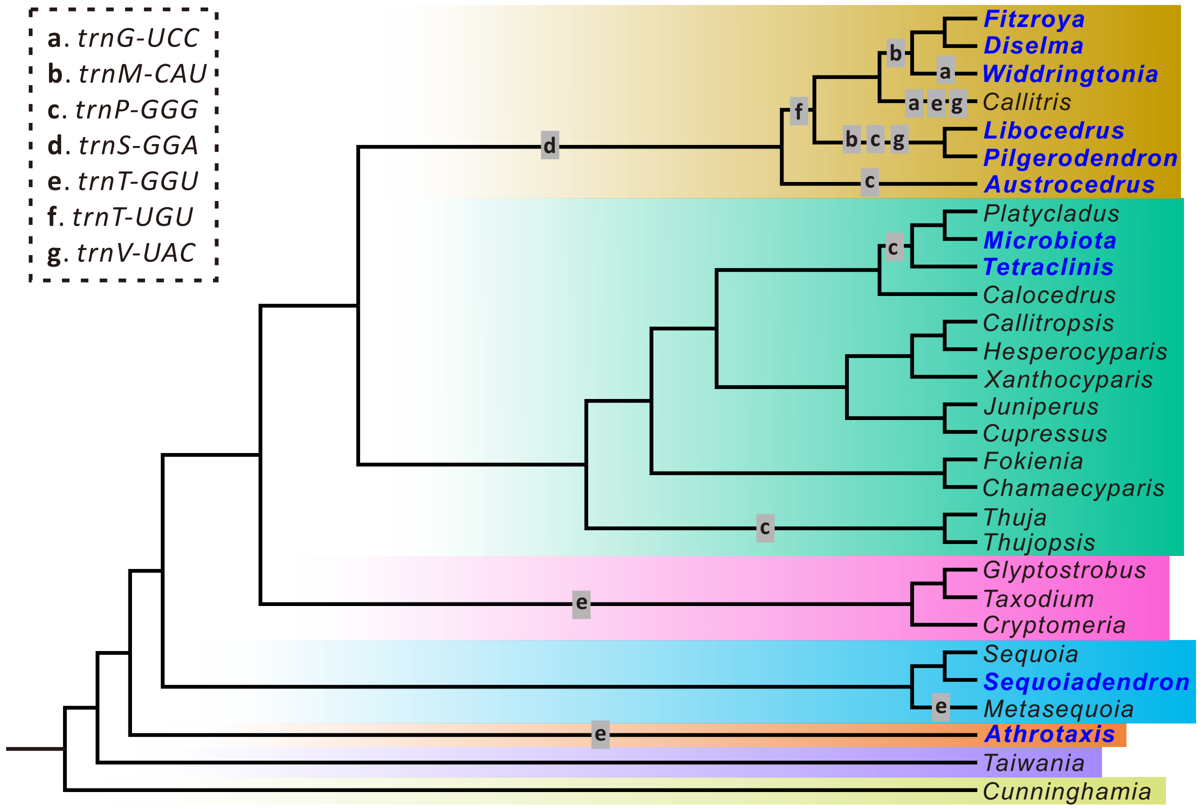

Figure S2. Summary of plastid tRNA gene loss events across the Cupressaceae phylogeny. Letters within gray bars denote the loss of the tRNAs indicated on the left side.

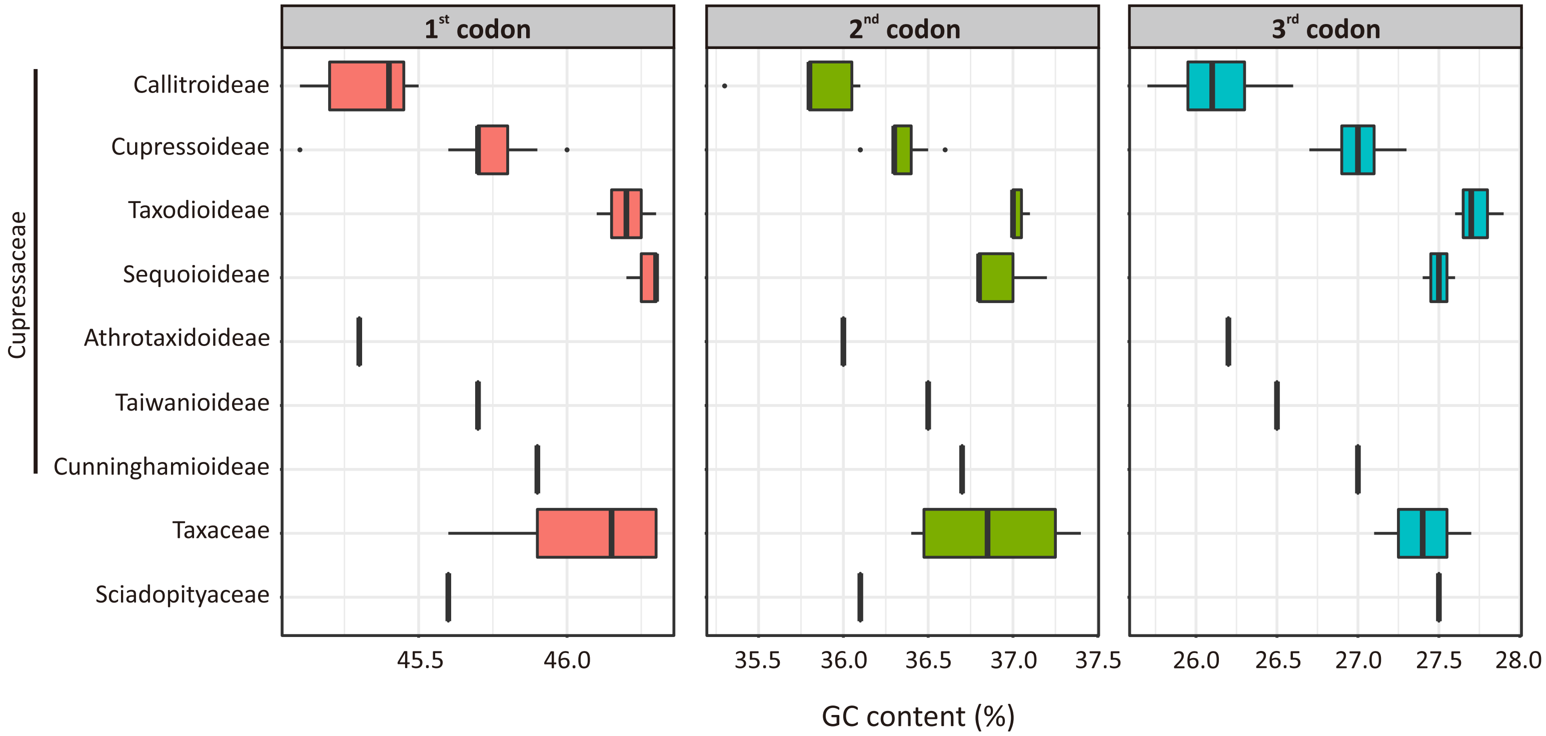

Figure S3. Comparison of the coding GC content. The GC content (percentage) at the 1st, 2nd, and 3rd codon positions were compared among the 29 sampled Cupressaceous genera and five closely related genera (one Sciadopityaceae and four Taxaceae).

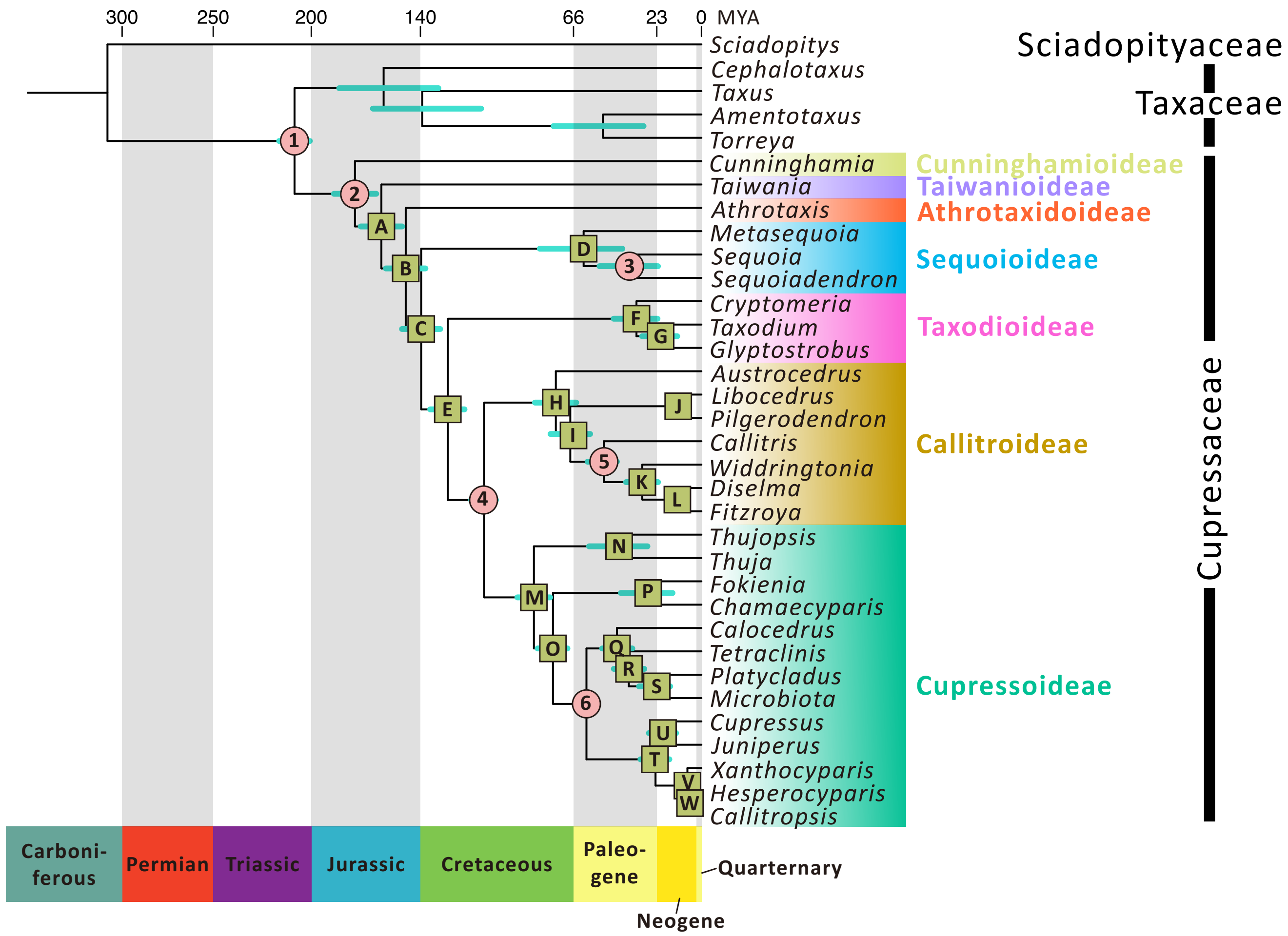

Figure S4. Molecular dating for the 29 sampled Cupressaceous genera and five closely related genera. Nodes 1–6 are constrained points. The estimated divergent times of the A–W nodes are shown in Table S2. Horizontal blue bars along nodes represent 95% confidence interval. MYA, million years ago.
