# Supplementary material for: The Origin and Evolution of Plastid Genome Downsizing in Southern Hemispheric Cypresses (Cupressaceae)
Source: Front Plant Sci. 2020 Jun 23;11:901. doi: 10.3389/fpls.2020.00901 (PMC7324783; doi:10.3389/fpls.2020.00901)
Supplement: Supplementary file 2 [file Data_Sheet_2.PDF]

Table S1. Collection localities, voucher information, and plastome GenBank accession numbers of the sequenced tree species.

Species	Collection locality	Voucher/tree number[a]	GenBank accession no.
**Athrotaxidoideae**			
Athrotaxis laxifolia Hook.	University of California Botanical Garden at Berkeley	Chaw1542/2012.0753	LC500575
**Sequoioideae**			
Sequoiadendron giganteum (Lindl.) J. Buchholz	University of California Botanical Garden at Berkeley	Chaw1538/2002.1062	LC500582
**Callitroideae**			
Austrocedrus chilensis (D. Don) Pic. Serm. & Bizzarri	University of California Botanical Garden at Berkeley	Chaw1548/83.0802	LC500576
Diselma archeri Hook. f.	University of California Botanical Garden at Berkeley	Chaw1544/65.0170	LC500577
Fitzroya cupressoides (Molina) I. M. Johnst.	University of California Botanical Garden at Berkeley	Chaw1547/2007.0165	LC500578
Libocedrus plumose (D. Don) Sarg.	University of California Botanical Garden at Berkeley	Chaw1550/90.0647	LC500579
Pilgerodendron uviferum (D. Don) Florin	University of California Botanical Garden at Berkeley	Chaw1546/2001.0044	LC500581
Widdringtonia schwarzii (Marloth) Mast.	University of California Botanical Garden at Berkeley	Chaw1540/96.0121	LC500584
**Cupressoideae**			
Microbiota decussata Kom.	Botanischer Garten der Heinrich-Heine-Universität Düsseldorf	Chaw1572/N.A.	LC500580
Tetraclinis articulate (Vahl) Mast	University of California Botanical Garden at Berkeley	Chaw1549/88.1295	LC500583

[a] Tree number refers to the specimen number in the cited Botanical Garden; N.A.: Not available

Table S2. Divergence times and their 95% confidence intervals estimated from the 34 sampled genera of Cupressales as depicted in Figure S6.

Node	Divergence time (MYA)	95% confidence interval
1*	209.95	202.01–212.87
2*	178.76	167.78–189.61
3*	37.10	22.42–52.58
4*	112.07	105.98–118.84
5*	50.33	43.59–58.76
6*	59.32	55.25–63.71
A	165.12	154.38–175.81
B	152.57	142.39–162.88
C	144.57	134.77–154.51
D	60.78	40.80–83.42
E	130.86	122.23–139.95
F	33.52	22.65–45.32
G	21.06	12.48–30.60
H	75.12	64.59–85.87
I	67.66	57.41–77.86
J	11.97	6.95–17.49
K	30.50	22.37–38.9
L	12.40	7.30–17.92
M	86.43	78.05–94.96
N	42.47	27.84–57.96
O	76.60	69.11–84.54
P	27.45	14.90–41.56
Q	43.61	35.64–51.22
R	37.45	29.46–45.21
S	24.06	16.09–32.17
T	23.69	16.52–31.46
U	19.82	13.05–27.10
V	7.23	4.62–10.13
W	5.87	3.44–8.59

*constraint nodes (TimeTree, Hedges et al., 2015)

MYA: million years ago

Table S3. Summary of predicted codon reassignment in the 34 sampled genera of Cupressales as outputted by CoreTracker

Predicted codon reassignment[a]	Genera	Prediction probability	Validation[b]
**CUC (L→I)**	*Calocedrus*	0.541	None
	Platycladus	0.593	None
	Callitris	0.592	None
	Sciadopitys	0.592	None
**CUU (L→I)**	*Taxus*	0.503	None
	Calocedrus	0.532	None
	Platycladus	0.583	None
	Callitris	0.582	None
	Sciadopitys	0.606	None
**CGC (R→Q)**	*Amentotaxus*	0.59	None
	Sciadopitys	0.59	None
**CUA (L→M)**	*Pilgerodendron*	0.588	None
	Callitris	0.537	None
	Libocedrus	0.588	None
**AUA (I→L)**	*Cunninghamia*	0.57	None
	Sequoia	0.601	None
**CUU (L→M)**	*Callitris*	0.531	None
**CUA (L→I)**	*Taxus*	0.509	None
	Calocedrus	0.54	None
	Platycladus	0.589	None
	Callitris	0.631	None
	Sciadopitys	0.631	None
**AGC (S→N)**	*Taxus*	0.593	None
	Torreya	0.593	None
	Glyptostrobus	0.593	None
	Cryptomeria	0.542	None
	Amentotaxus	0.57	None
	Cephalotaxus	0.541	None
	Sciadopitys	0.626	None
	Taiwania	0.542	None
**CGC (R→K)**	*Athrotaxis*	0.589	None
	Diselma	0.536	None
	Taxus	0.577	None
	Callitris	0.588	None
	Sciadopitys	0.577	None
**AGG (R→K)**	*Austrocedrus*	0.59	None
	Athrotaxis	0.592	None
	Diselma	0.537	None
	Taxus	0.719	None
	Torreya	0.723	None
	Cunninghamia	0.666	None
	Widdringtonia	0.538	None

	Cryptomeria	0.538	None
	Metasequoia	0.591	None
	Amentotaxus	0.766	None
	Sequoiadendron	0.537	None
	Cephalotaxus	0.729	None
	Callitris	0.594	None
	Sciadopitys	0.769	None
	Taiwania	0.7	None
**UUA (L→I)**	*Calocedrus*	0.525	None
	Callitris	0.577	None
	Sciadopitys	0.575	None
**GAC (D→E)**	*Callitris*	0.533	None
**AGA (R→K)**	*Austrocedrus*	0.577	None
	Athrotaxis	0.577	None
	Diselma	0.525	None
	Taxus	0.68	None
	Torreya	0.681	None
	Cunninghamia	0.579	None
	Widdringtonia	0.525	None
	Cryptomeria	0.526	None
	Metasequoia	0.577	None
	Amentotaxus	0.657	None
	Sequoiadendron	0.526	None
	Cephalotaxus	0.681	None
	Callitris	0.577	None
	Sciadopitys	0.721	None
	Taiwania	0.577	None
**CGU (R→K)**	*Taxus*	0.563	None
	Torreya	0.563	None
	Cunninghamia	0.576	None
	Amentotaxus	0.574	None
	Cephalotaxus	0.563	None
	Sciadopitys	0.562	None
	Taiwania	0.576	None
**UUA (L→M)**	*Pilgerodendron*	0.577	None
**GAG (E→D)**	*Fokienia*	0.517	None
	Tetraclinis	0.533	None
	Callitris	0.615	None
**CGA (R→K)**	*Austrocedrus*	0.583	None
	Athrotaxis	0.582	None
	Diselma	0.532	None
	Taxus	0.569	None
	Torreya	0.569	None
	Widdringtonia	0.532	None
	Cryptomeria	0.531	None
	Metasequoia	0.582	None
	Amentotaxus	0.58	None

	Sequoiadendron	0.531	None
	Cephalotaxus	0.569	None
	Callitris	0.582	None
	Sciadopitys	0.569	None
**CUG (L→M)**	*Austrocedrus*	0.593	None
	Pilgerodendron	0.593	None
	Callitris	0.54	None
	Libocedrus	0.593	None
**AGG (R→Q)**	*Taxus*	0.547	None
	Amentotaxus	0.591	None
**CGG (R→K)**	*Diselma*	0.54	None
	Taxus	0.581	None
	Torreya	0.581	None
	Cunninghamia	0.592	None
	Widdringtonia	0.54	None
	Cryptomeria	0.54	None
	Amentotaxus	0.59	None
	Callitris	0.592	None
	Sciadopitys	0.581	None
**AGA (R→Q)**	*Taxus*	0.572	None
	Torreya	0.576	None
	Amentotaxus	0.576	None
	Sciadopitys	0.551	None
**CGA (R→Q)**	*Taxus*	0.581	None
	Torreya	0.583	None
	Amentotaxus	0.583	None
	Sequoiadendron	0.517	None
	Sciadopitys	0.662	None
**AGU (S→N)**	*Taxus*	0.583	None
	Torreya	0.583	None
	Glyptostrobus	0.583	None
	Cryptomeria	0.532	None
	Amentotaxus	0.561	None
	Cephalotaxus	0.532	None
	Sciadopitys	0.583	None
	Taiwania	0.533	None
**UUG (L→M)**	*Pilgerodendron*	0.582	None
	Callitris	0.531	None
	Libocedrus	0.582	None
**CGU (R→Q)**	*Taxus*	0.574	None
**CUG (L→I)**	*Callitris*	0.592	None
	Sciadopitys	0.59	None
**UUG (L→I)**	*Callitris*	0.583	None
	Sciadopitys	0.581	None
**CGG (R→Q)**	*Taxus*	0.591	None
	Torreya	0.593	None
	Amentotaxus	0.593	None

Sequoiadendron	0.524	None
Sciadopitys	0.628	None

[a]Predicted codon reassignment notation:
codon (original amino acid → reassigned amino acid)
e.g. CUC (L→I) indicates CUC codon usually encodes Leucine (L), but may be reassigned to encode Isoleucine (I).

[b]Validation step of CoreTracker. “None” indicates that both clade-aware validation and the validation based on alignment improvement are not met.
